# Association between migraine and cardiovascular disease: A cross-sectional study

**DOI:** 10.3389/fcvm.2022.1044465

**Published:** 2022-11-24

**Authors:** Kai Wang, Yukang Mao, Miao Lu, Yinzhang Ding, Zhongming Li, Yansong Li, Xianling Liu, Yan Sun, Jian Hong, Di Xu, Tingting Wu

**Affiliations:** ^1^Department of Geriatrics, The First Affiliated Hospital of Nanjing Medical University, Nanjing, Jiangsu, China; ^2^Department of Cardiology, The First Affiliated Hospital of Nanjing Medical University, Nanjing, Jiangsu, China; ^3^Department of Cardiology, Suzhou Municipal Hospital, Gusu School, The Affiliated Suzhou Hospital of Nanjing Medical University, Nanjing Medical University, Suzhou, Jiangsu, China

**Keywords:** migraine, cardiovascular disease, adults, cross-sectional study, National Health and Nutrition Examination Survey (NHANES)

## Abstract

**Background:**

Cardiovascular disease (CVD) poses a tremendous threat to global health, giving rise to exceedingly high morbidity and mortality among patients. A migraine is a common neurological disorder characterized by recurrent attacks of severe headache, while its cardiovascular burden remains unclear. Therefore, this study aims to investigate whether migraine is associated with CVD.

**Methods:**

The cross-sectional data of 5,692 subjects aged 20 or above was obtained from the National Health and Nutrition Examination Survey (NHANES) 1999–2004. To determine whether migraine is associated with CVD, weighted logistic regression models were used. In a subsequent subgroup analysis, several confounding factors were also explored to investigate the association between migraine and CVD.

**Results:**

In total, 5,692 subjects were enrolled in this study, with the prevalence of CVD being 13.3%. Participants with CVD tended to be older, male, non-Hispanic whites, more educated, former smokers, and alcohol drinkers, and had a higher waist circumference, less physical activity, a higher level of triglyceride and creatinine as well as a lower level of high-density lipoprotein cholesterol (HDL-C) and estimated glomerular filtration rate (eGFR) (all *P* < 0.05). Considering all potential confounders, migraine was associated with a higher risk of CVD [odds ratios (ORs) 2.77; 95% confidence intervals (CIs): 1.56–4.90]. Subgroup analysis showed a higher risk of CVD in females, those older than 60 years, with a lower body mass index (BMI) level (≤ 30 kg/m^2^), a higher level of eGFR (> 90 mL/min/1.73 m^2^), hypertension and hyperlipidemia and without diabetes.

**Conclusion:**

In summary, our study revealed a positive association between migraine with CVD in a nationally representative US adult population. Our findings highlighted that migraine should be considered an important risk factor for CVD.

## Introduction

According to a recent report from the American Heart Association, the global prevalence of cardiovascular diseases (CVD) has been increasing exponentially during the past few decades, leading to significantly increased disability and premature mortality among patients, posing a threat to public health worldwide (1). Although traditional risk factors for CVD such as hypertension, diabetes, and obesity are gaining more attention than before, the persistent prevalence of CVD indicates a great gap in our knowledge on CVD risk factors (1). Therefore, the exploitation of novel CVD risk factors is urgently needed to prevent the progression of CVD.

Migraine is a common and disabling neurological disorder with unknown etiology which adversely affects the quality of life of approximately one billion people across the world (2), and whose primary clinical manifestations can be simply generalized as recurrent attacks of severe headache accompanied by a series of concomitant autonomic symptoms, including nausea, vomiting, photophobia and phonophobia (3). The global prevalence of migraine during a lifetime varies between 10–20%, largely depending on the disease’s diagnostic criteria and the age and gender differences among target populations. Typically, the incidence of migraine increases with age, peaks in young and middle-aged adulthood, and tends to be much higher in women than men (4, 5).

The strong association between migraine and major cardiovascular diseases (CVDs) that damage human health, such as ischemic and hemorrhagic stroke, ischemic heart disease (IHD), myocardial infarction (MI), and atrial fibrillation (AF), has been convincingly demonstrated by several large population-based cohort studies conducted across the world, especially among female migraineurs and migrainers with aura (6–12). From the perspective of pathophysiology, the underlying mechanisms linking migraine with CVD may include endothelial dysfunction, restricted vasodilation, excessive platelet activation, aggregation, spreading depolarization, shared genetic mutations, common comorbidities [such as patent foramen ovale (PFO)], and the use of non-steroidal anti-inflammatory drugs (13–23). Since a comprehensive understanding of migraine-associated cardiovascular morbidity can facilitate the self-management and medical practice of migraine, this study aims to explore the association between migraine and CVD using data from the National Health and Nutrition Examination Surveys (NHANES).

## Materials and methods

### Study population

**FIGURE 1:**
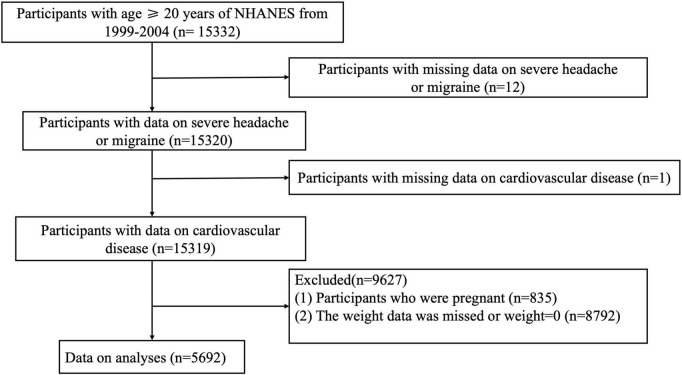
Detailed information about subject recruitment.

This article was based on publicly-available data from the NHANES. It is available on the Centers for Disease Control and Prevention National Center for Health Statistics^1^. Our study originally comprised subjects above 20 years with available data on migraine and CVD from the NHANES 1999–2004 (*n* = 15319). After excluding pregnant women (*n* = 835) and individuals with missing or invalid weight data (*n* = 8,792), 5,692 subjects were ultimately enrolled for further analyses. NHANES Institutional Review Board approved the ethical conduct of NHANES 1999–2004 (Protocol#98-12), and all participants provided informed consent. The study procedures were structured in line with the Declaration of Helsinki. Detailed information about subject recruitment is illustrated in Figure 1.

### Assessment of migraine

Participants who provided a previous diagnosis of migraine and answered “yes” to the following question during the interview: “During the past three months, did you have severe headaches or migraines?” were considered migraineurs. Clinically, there are two primary subtypes of migraine: migraine without aura (MO) and migraine with aura (MA). Generally, MO differs from MA largely due to predisposing factors and no apparent prodromal symptoms, most notably transient focal neurological symptoms. MA is less associated with other conditions with known effects in inducing headache and is commonly preceded or sometimes accompanied by a series of prodromal symptoms, including emotional fluctuations, loss of appetite, insomnia, spiritlessness, and neck stiffness and pain. We accessed migraine based on the Third Edition of the International Classification of Headache Disorders (ICHD-3). Additionally, although the NHANES database lacks explicit information about migraine subtypes, given that most migraineurs present with similar symptoms, this study will most likely show that most subjects develop MO (3).

### Assessment of cardiovascular disease outcomes

The CVD outcomes were self-reported diagnoses of five major CVD events, including congestive heart failure (CHF), coronary heart disease (CHD), angina pectoris, heart attack, and stroke. Those who answered “yes” to the following question: “Have you ever been told by a physician that you had CHD/CHF/angina/a heart attack or a stroke?” was labeled as having CVD. However, use of self-reported measures are prone to recall bias, which may have an impact on the interpretation of the data.

### Covariates

Biological considerations and published works in the literature were used to select covariates. Using a standardized questionnaire, we collected each participant’s demographic characteristics, including age, sex, educational level, race/ethnicity, family income to poverty (PIR) ratio, physical activity, smoking status, alcohol consumption, diabetes history, and hypertension history, and hyperlipidemia history. Each subject’s height, weight, and waist circumference were measured during physical examination, and the body mass index (BMI) was accordingly calculated. Serum concentrations of total cholesterol (TC), triglyceride (TG), low-density lipoprotein cholesterol (LDL-C), high-density lipoprotein cholesterol (HDL-C), and creatinine were examined by laboratory tests.

We classified education levels into three groups: below high school, high school, and above high school. The following categories were used to classify race and ethnicity: Mexican American, non-Hispanic black, non-Hispanic white, other Hispanic, and other race groups. A family PIR below one indicates poverty. Participants’ smoking status was categorized into three groups: now, former, and non-smokers. They were considered alcohol consumers if they consumed at least 12 drinks of alcohol during the past 12 months (24). Standardized physical and laboratory examinations were conducted. Work activity was defined as physical activity. BMI was calculated as weight in kilograms (kg) divided by the square of height in meters (m^2^) (25). Diagnostic criteria for diabetes were as follows: (1) an HbA1c level exceeding 6.5% (47.5 mmol/mol) was indicated; (2) there was a fasting plasma glucose (FPG) level of 126 mg/dL (7.0 mmol/L) or higher; (3) blood glucose was 200 mg/dl (11.1 mmol/L) or higher at random; (4) The plasma glucose level at the end of a 2-h oral glucose tolerance test (OGTT) was greater than or equal to 200 mg/dl (11.1 mmol/L); (5) a hypoglycemic drug’s use; or (6) participants who self-reported having diabetes (26). According to these criteria, hypertension is defined as one of six criteria: (1) hypertension diagnosed by medical practitioners based on their self-reports; (2) antihypertensive medication use based on self-report; (3) A minimum of three times on different days, the systolic blood pressure (SBP) ≥ 140 mmHg; (4) a minimum of three times on different days, the diastolic blood pressure (DBP) ≥ 90 mmHg; (5) average systolic blood pressure (SBP) ≥ 140 mmHg; and (6) average diastolic blood pressure (DBP) ≥ 90 mmHg. Based on the Physician Examination Procedures Manual from the National Center for Health Statistics (August 2000, available at:)^2^, average blood pressure was calculated: (1) those DBP readings displaying zero were not included in the calculation of the average DBP, and the average DBP was recorded only when all DBP readings were zero; (2) the average blood pressure was determined if only one reading were valid; and (3) a blood pressure average is always calculated excluding the first reading if there was more than one. The following definition was used to define hyperlipidemia: (1) TG ≥ 150mg/dl (1.7 mmol/L); (2) TC ≥ 200mg/dl (5.18 mmol/L); (3) LDL ≥ 130 mg/dl (3.37 mmol/L); (4) HDL < 40 mg/dl (1.04 mmol/L) for male, HDL < 50 mg/dl (1.3 mmol/L) for female; (5) participants who used lipid-lowering medication; or (6) those with a self-reported hyperlipidemia diagnosis (27). Estimated glomerular filtration rate (eGFR) was estimated using an equation initiated by the Chronic Kidney Disease Epidemiology Collaboration (CKD-EPI) with high efficacy and accuracy: eGFR = 141 × min (Scr/k,1)*^a^* × max (Scr/k,1)^–1^.^209^ × 0.993*^Age^* × 1.018 (if female) × 1.159 (if black). In this formula, Scr refers to serum creatinine, *k* is 0.9 for males and 0.7 for females, a is −0.411 for males and −0.329 for females, min is minimum of Scr/k or 1, and max is maximum of Scr/k or 1 (28). A detailed description of these covariates can be found on the NHANES website.

### Statistical analysis

Our study considered complex sampling designs and weights according to the NHANES analytic guidelines (29). All analyses were performed using the weights of the fasting subsample. Due to significant differences between sampling designs for 1999–2002 and 2003–2004, we separated the data for 1999–2004 into two groups. Consequently, we calculated the sampling weights according to the formula: 1999–2004 weights = 2/3 of the 1999–2002 weight or 1/3 of the 2003–2004 weight. In categorical variables, frequency is expressed as a percentage, while continuous variables are expressed as median (interquartile range, IQR) or mean (standard deviation, SD). Based on the baseline characteristics of the subjects with migraine and those without migraine, a student’s *t*-test or a non-parametric test was applied to analyze the statistical difference for the continuous variables, and a χ^2^-square test was used to analyze categorical variables.

A logistic regression model investigated the relationship between migraine and CVD. We tested regression models (Models 1 to 3) by adjusting potential confounding factors incrementally. Model 1 was adjusted for age, sex, educational level, and race. Model 2 was further adjusted for poverty, physical activity, smoking status, alcohol intake, BMI, waist circumference, diabetes, hypertension, and hyperlipidemia. Model 3 was adjusted for the variables in Model 2 and additional confounders, including TC, TG, LDL-C, HDL-C, and eGFR. A 95% confidence interval (CI) and adjusted odds ratio (OR) were presented in the results. Following this, subgroup analyses were conducted stratified by age, sex, BMI, eGFR, hypertension, diabetes, and hyperlipidemia to examine the association between migraine and CVD in further detail.

The statistical analyses were conducted using the statistical package R version 4.1.3. It was considered statistically significant when the *P*-value < 0.05.

## Results

### Baseline characteristics of subjects

Table 1 provides detailed baseline information for all subjects in this study. A total of 5,692 subjects were included in this study, of whom 1090 (19.1%) had migraines or severe headaches. The overall prevalence of CVD was 13.3%. Participants with CVD tended to be older, male, non-Hispanic whites, more educated, former smokers, and non-alcohol drinkers, and had a higher waist circumference, less physical activity, a higher level of TG and creatinine as well as a lower level of HDL-C and eGFR (all *P* < 0.05). Although lower eGFR levels have been consistently noted in CVD group compared with non-CVD group during the early and middle stages of kidney disease (eGFR ≥ 30 ml/min/1.73 m^2^), difference in eGFR levels were not statistically significant between the two during the end stage of kidney disease (eGFR < 30 ml/min/1.73 m^2^). In addition, participants with CVD were more likely to develop diabetes, hypertension, and hyperlipidemia. The baseline characteristics of participants included and excluded are shown in Supplementary Table 1.

**TABLE 1:** Baseline characteristics of the study participants (weighted).

	Total	Non-CVD	CVD	*P*-*value*
Participants (n)	5,692	4,935	757	
Age, years [mean(95%CI)]	51.85 (51.08,52.62)	48.96 (48.15,49.76)	69.79 (68.66,70.92)	<0.001
Sex				0.020
Female	2,893 (50.85) [48.09,53.62]	2,543 (51.74) [50.27,53.21]	350 (45.36) [40.20,50.51]	
Male	2,799 (49.15) [46.48,51.81]	2,392 (48.26) [46.79,49.73]	407 (54.64) [49.49,59.80]	
Education				<0.001
Below high school	951 (16.60) [14.04,19.16]	767 (15.06) [12.73,17.39]	184 (26.75) [22.40,31.10]	
High school	2,302 (40.30) [37.66,42.95]	1,964 (39.90) [37.73,42.08]	338 (44.18) [39.54,48.81]	
Above high school	2,418 (42.63) [39.57,45.70]	2,189 (45.04) [42.51,47.56]	229 (29.08) [24.88,33.28]	
Race				<0.001
Mexican American	1,194 (20.98) [16.01,25.94]	1075 (21.91) [17.23,26.59]	119 (15.16) [8.89,21.44]	
Non-Hispanic Black	1,083 (18.81) [15.51,22.10]	972 (19.56) [15.96,23.16]	111 (14.12) [10.80,17.44]	
Non-Hispanic White	2,954 (51.76) [46.34,57.18]	2467 (49.65) [44.98,54.31]	487 (64.90) [58.78,71.01]	
Other Hispanic	263 (4.60) [2.65,6.56]	245 (4.97) [2.89,7.05]	18 (2.31) [0.92,3.70]	
Other Race	198 (3.85) [3.00,4.70]	176 (3.90) [3.08,4.73]	22 (3.51) [2.00,5.03]	
Poverty				0.410
No	4,186 (73.64) [69.73,77.56]	3635 (81.61) [79.85,83.38]	551 (82.93) [79.50,86.36]	
Yes	923 (16.39) [14.58,18.20]	802 (18.39) [16.62,20.15]	121 (17.07) [13.64,20.50]	
BMI, kg/m^2^ [mean (95%CI)]	28.43 (28.19,28.66)	28.40 (28.13,28.66)	28.66 (28.07,29.25)	0.46
Waist, cm [mean (95%CI)]	97.45 (96.88,98.01)	96.84 (96.21, 97.47)	101.72 (100.24,103.21)	<0.001
TC, mmol/l [mean (95%CI)]	5.22 (5.17,5.27)	5.23 (5.18,5.29)	5.15(5.05,5.25)	0.150
Triglyceride, mmol/l [median (IQR)]	1.37 (0.96,2.05)	1.32 (0.94,2.00)	1.68 (1.14,2.42)	<0.001
LDL-C, mmol/l [mean (95%CI)]	3.14 (3.08,3.20)	3.16 (3.09,3.22)	2.99 (2.84,3.14)	0.060
HDL-C, mmol/l [mean (95%CI)]	1.35 (1.33,1.36)	1.35 (1.33,1.37)	1.30 (1.26,1.34)	0.040
Creatinine, umol/l [median (IQR)]	70.72 (61.88,88.40)	70.72 (61.88, 88.40)	88.40 (70.70,106.08)	<0.001
eGFR, mL/min/1.73m^2^ [mean (95%CI)]				
Total	90.92 (89.64,92.21)	94.10 (92.90,95.30)	69.15 (67.01,71.29)	<0.001
eGFR ≥ 90	108.62 (107.85,109.39)	108.93 (108.15,109.70)	101.96 (99.71,104.22)	<0.001
60 ≤ eGFR < 90	77.36 (76.78,77.94)	77.63 (77.05,78.21)	75.95 (74.74,77.17)	0.007
30 ≤ eGFR < 60	48.85 (47.97,49.73)	49.98 (49.09,50.87)	47.18 (45.64,48.71)	0.002
eGFR < 30	19.99 (16.76,23.23)	19.60 (14.12,25.08)	20.27 (16.55,24.00)	0.843
Physical activity				
No	2,678 (46.77) [43.78,49.76]	2199 (44.24) [41.50,46.98]	479 (63.24) [59.61,66.87]	<0.001
Yes	3,004 (53.02) [49.44,56.60]	2728 (55.76) [53.02,58.50]	276 (36.76) [33.13,40.39]	
Severe headache or migraine				0.220
No	4,602 (80.76) [77.12,84.39]	3966 (80.38) [78.70,82.06]	636 (83.11) [79.51,86.71]	
Yes	1,090 (19.24) [17.43,21.05]	969 (19.62) [17.94,21.30]	121 (16.89) [13.29,20.49]	
Smoking status				<0.001
Never	2,829 (48.88) [46.39,51.37]	2,521 (50.70) [48.43,52.97]	308 (38.60) [33.69,43.51]	
Former	1,573 (28.47) [26.06,30.89]	1,239 (25.83) [24.00,27.65]	334 (45.55) [41.08,50.02]	
Now	1,274 (22.35) [20.48,24.22]	1,162 (23.47) [21.65,25.29]	112 (15.85) [12.16,19.55]	
Alcohol intake				0.010
No	1,555 (26.99) [24.36,29.63]	1,339 (31.00) [28.41,33.59]	216 (37.29) [32.35,42.22]	
Yes	3,307 (57.89) [54.47,61.30]	2,925 (69.00) [66.41,71.59]	382 (62.71) [57.78,67.65]	
DM				<0.001
No	4,902 (85.43) [81.61,89.24]	4,383 (88.52) [87.13,89.91]	519 (66.43) [61.87,70.99]	
Yes	789 (14.54) [13.10,15.98]	551 (11.48) [10.09,12.87]	238 (33.57) [29.01,38.13]	
Hypertension				<0.001
No	3,091 (53.85) [50.97,56.73]	2,956 (59.69) [57.78,61.60]	135 (17.73) [12.96,22.50]	
Yes	2,599 (46.11) [43.12,49.10]	1,977 (40.31) [38.40,42.22]	622 (82.27) [77.50,87.04]	
Hyperlipidemia				<0.001
No	1,563 (28.08) [26.16,30.00]	1,445 (32.22) [30.58,33.86]	118 (18.78) [15.21,22.35]	
Yes	3,702 (64.06) [60.84,67.29]	3,164 (67.78) [66.14,69.42]	538 (81.22) [77.65,84.79]	

Data are shown as unweighted number (weighted percentage, %) (95% CI), weighted mean (95% CI) and weighted median (IQR). CVD, cardiovascular disease; BMI, body mass index; DM, diabetes mellitus; TC, total cholesterol; LDL-C, low-density lipoprotein cholesterol; HDL-C, high density leptin cholesterol; eGFR, estimated glomerular filtration rate; CI, confidence interval; IQR, interquartile range.

### Association of migraine with cardiovascular disease

As displayed in Table 2, sampling-weighted multivariable logistic regression analysis was used to examine the association between migraine and CVD. In all three regression models (Model 1-3), a higher risk of CVD was associated with migraine or severe headache (all *P* < 0.05). After fully adjusting for potential confounders, including age, sex, educational level, race, poverty, physical activity, smoking status, alcohol intake, BMI, waist circumference, diabetes, hypertension, hyperlipidemia, TC, TG, LDL-C, HDL-C, and eGFR, in participants with migraine or severe headaches, the ORs (95% CIs) for the overall prevalence of CVD were 2.77 (1.56–4.90) (*P* = 0.001). From the perspective of specific CVD, migraine or severe headache was significantly associated with the prevalence of angina (OR 2.27; 95% CI: 1.01–5.07) and stroke (OR 3.80; 95% CI: 1.54–9.38) (Table 3).

**TABLE 2:** Association between severe headache or migraine and CVD in the database from NHANES 1999-2004 (weighted).

	Unweighted number of total participants	Unweighted number of CVD participants	Model 1 OR (95% CI)	Model 2 OR (95% CI)	Model 3 OR (95% CI)
Without severe headache or migraine	4602	636	1 (Ref).	1 (Ref).	1 (Ref).
With severe headache or migraine	1090	121	1.82 (1.27-2.61)	1.87 (1.22-2.88)	2.77 (1.56-4.90)
*P*-value			0.002	0.006	0.001

Model 1 was adjusted for age, sex, race, and education. Model 2 was adjusted for Model 1 + poverty, physical activity, smoking status, alcohol intake, BMI, waist, DM, hypertension, and hyperlipidemia. Model 3 was adjusted for Model 2 + eGFR, triglyceride, total cholesterol, LDL-C, HDL-C.

NHANES, National Health and Nutrition Examination Survey; CVD, cardiovascular disease; BMI, body mass index; DM, diabetes mellitus; LDL-C, low-density lipoprotein cholesterol; HDL-C, high density leptin cholesterol; eGFR, estimated glomerular filtration rate; OR, odds ratio; CI, confidence interval.

**TABLE 3:** Adjusted odds ratios (95% CI) for association between severe headache or migraine and individual CVDs.

	CHF	CHD	Angina	Heart attack	Stroke
	
	OR (95% CI)	OR (95% CI)	OR (95% CI)	OR (95% CI)	OR (95% CI)
Without severe headache or migraine	1 (Ref).	1 (Ref).	1 (Ref).	1 (Ref).	1 (Ref).
With severe headache or migraine	1.85 (0.52-6.60)	0.80 (0.37-1.77)	2.27 (1.01-5.07)	1.33 (0.52-3.44)	3.80 (1.54-9.38)
*P*-value	0.323	0.570	0.046	0.533	0.006

Analyses were adjusted for age, sex, race, and education, poverty, physical activity, smoking status, alcohol intake, BMI, waist, DM, hypertension, hyperlipidemia, eGFR, triglyceride, total cholesterol, LDL-C and HDL-C. CVD, cardiovascular disease; CHF, congestive heart failure; CHD, coronary heart disease; BMI, body mass index; DM, diabetes mellitus; LDL-C, low-density lipoprotein cholesterol; HDL-C, high density leptin cholesterol; eGFR, estimated glomerular filtration rate; OR, odds ratio; CI, confidence interval.

### Subgroup analysis

As illustrated in Table 4, subgroup analysis based on age, sex, BMI, eGFR, hypertension, diabetes and hyperlipidemia was performed. Statistically significant association between migraine or severe headache and CVD was found in subgroup analyses stratified by sex, eGFR, and diabetes (all *P* for interaction < 0.05). Participants who were older than 60 years, female, had a lower BMI level (≤ 30 kg/m^2^), a higher level of eGFR (> 90 ml/min/1.73 m^2^), hypertension and hyperlipidemia and had no co-existed diabetes were prone to have a higher risk of CVD.

**TABLE 4:** Subgroups analysis for the associations of severe headache or migraine and CVD (weighted).

Subgroups	Without severe headache or migraine (OR)	With severe headache or migraine [OR (95% CI)]	*P*-*value*	*P* for interaction
Age				0.449
≤ 60 years	1 (Ref).	2.20 (0.84-5.77)	0.103	
> 60 years	1 (Ref).	2.69 (1.01-7.19)	0.049	
Sex				<0.001
Male	1 (Ref).	9.75 (0.41-2.31)	0.952	
Female	1 (Ref).	6.02 (2.59-13.95)	<0.001	
BMI, kg/m^2^				0.684
BMI ≤ 30	1 (Ref).	2.57 (1.32-5.00)	0.008	
BMI > 30	1 (Ref).	3.19 (0.88-1.16)	0.074	
BMI > 30	1 (Ref).	3.19 (0.88-11.52)	0.074	
eGFR, mL/min/1.73m^2^				<0.05
eGFR ≤ 90	1 (Ref).	1.51 (0.69-3.33)	0.287	
eGFR > 90	1 (Ref).	3.54 (1.38-9.10)	0.011	
Hypertension				0.109
Yes	1 (Ref).	3.57 (1.80-7.07)	<0.001	
No	1 (Ref).	1.35 (0.41-4.41)	0.608	
Diabetes mellitus				0.042
Yes	1 (Ref).	0.38 (0.06-2.42)	0.275	
No	1 (Ref).	3.50 (1.86-6.60)	< 0.001	
Hyperlipidemia				0.972
Yes	1 (Ref).	2.74 (1.45-5.18)	0.003	
No	1 (Ref).	4.81 (0.59-3.92)	0.134	

Analyses was adjusted for age, sex, race, and education, poverty, physical activity, smoking status, alcohol intake, BMI, waist, diabetes mellitus, hypertension, hyperlipidemia, eGFR, triglyceride, total cholesterol, LDL-C and HDL-C. CVD, cardiovascular disease; BMI, body mass index; LDL-C, low-density lipoprotein cholesterol; HDL-C, high density leptin cholesterol; eGFR, estimated glomerular filtration rate; OR, odds ratio; CI, confidence interval.

## Discussion

Our cross-sectional study included data from 5,692 subjects participating in NHANES 1999–2004 for analysis. There was a positive association between migraine or severe headache and CVD, independent of other confounding factors, such as age and gender, race, education, poverty, physical activity, smoking status, alcohol intake, BMI, waist, diabetes mellitus, hypertension, hyperlipidemia, eGFR, triglyceride, total cholesterol, LDL-C, and HDL-C, in a nationally representative US population. In the fully adjusted model, there was a higher risk for CVD among participants with migraines or severe headaches, with ORs and 95% CIs of 2.77 (1.56–4.90). Subsequent subgroup analysis indicates that ORs for the association of migraine or severe headache with CVD were higher among migraineurs who were older than 60 years, female, with a lower BMI level (≤ 30 kg/m^2^), a higher level of eGFR (> 90 ml/min/1.73 m^2^), hypertension and hyperlipidemia and without diabetes.

Although the overall prevalence of CVD was found to be positively correlated with migraine in this study, we primarily focused on the association of migraine with angina and stroke, supported by several recent studies. According to a Danish population-based cohort study, migraine patients were found to have a higher risk of MI (OR 1.49, 95% CI: 1.36–1.64), ischemic stroke (OR 2.26, 95% CI: 2.11–2.41), and hemorrhagic stroke (OR 1.94, 95% CI: 1.68–2.23) after an 18-year follow-up (11), which was in line with the findings in the Nurses’ Health Study II of 115541 young- and middle-aged female nurses that migraine was positively associated with MI (OR 1.39, 95% CI: 1.18–1.64), stroke (OR 1.62, 95% CI: 1.37–1.92), angina/coronary revascularization (OR 1.73, 95% CI: 1.29–2.32), and cardiovascular mortality (OR 1.37, 95% CI: 1.02–1.83) (10). A recently published meta-analysis of 18 prospective cohort studies incorporating 1.6 million migraineurs documented that migraine was associated with a greater risk of MI and stroke (including unspecified, ischemic, and hemorrhagic) and that MA was associated with a higher risk of cardiovascular mortality, with a moderate to severe degree of heterogeneity for the cardiovascular outcomes existing in these studies being fundamentally attributed to the presence of aura (30). Generally speaking, estimated ORs in this study were much higher than those in previous studies. A reasonable explanation for these differences may lie in that we included more patients that suffered from severe migraine than in previous studies, as anyone who possessed a self-reported diagnosis of migraine by a physician may have at least experienced one hospital visit, probably due to higher frequency, longer duration or unbearable pain of headache attack. This evidence implies the strong association between migraine and cardio- and cerebrovascular events.

During the past few decades, although extensive published literature has reported the association between migraine and CVD, the underlying mechanisms appear to be complex and multi-dimensional. From the perspective of pathophysiology, the association between migraine and CVD may reflect the possibility of common cellular and molecular basis underlying the pathogenesis of both diseases. A downregulated number and function of endothelial progenitor cells, endothelial dysfunction, restricted vasoconstriction, platelet aggregation, and overactivation of coagulation cascade are key events that are closely involved with the pathogenesis of migraine, and these factors also have a critical role in accelerating CVD development (13–16). The genome-wide analysis determined a considerable overlap between migraine and CVD on a genetic basis, especially coronary heart disease (CAD) and ischemic stroke (18, 19). Some drugs used to treat migraines, such as non-steroidal anti-inflammatory drugs, triptans, and ergotamine, can exert adverse effects on cardiovascular function, which appears to be further impaired by the increasing dosage and degree of drug intake (22, 31, 32). In addition, migraine has been strongly linked with well-established risk factors for CVD, such as hypertension, diabetes, and hypercholesterolemia, which is more frequently observed among migraineurs with aura, suggesting that migraine may contribute to the development of CVD, at least in part by increasing other cardiovascular risk profiles (33, 34). In a large-scale prospective study of Swedish young adults, Nyberg et al. demonstrated that subjects with a lower cardiovascular fitness were more susceptible to migraine that requires pharmacological intervention (35). Interestingly, PFO has emerged as a novel mechanism connecting migraine and CVD recently, which can be a leading cause of paradoxical embolism and subsequent coronary and cerebral ischemic events, and whose prevalence is significantly higher among migraineurs with aura (47%) than those without aura (16%) (21, 36).

Although the impact of a migraine-associated increase in cardiovascular risk seems negligible at the individual level, it may accumulate and transfer into a considerable CVD risk at the population level, given the high prevalence of migraine in the general population. In this case, the QRISK3 risk prediction models that incorporate migraine as an indicator for cardiovascular risk stratification have been developed and implemented to estimate the risk of CVD in the following ten years among adult male and female residents, indicating that the emerging recognition of migraine is an important risk factor for CVD to be taken into account and that it is crucial to identify those high-risk migraineurs for CVD in clinical practice (37). Besides the early detection and identification of CVD process in migraine patients, timely pharmacological interventions may also be in favor of improving the prognosis of migraine patients with CVD. Although aspirin or clopidogrel has not been explicitly recommended in treating migraine (38, 39), medical practitioners should re-consider whether migraine patients at a particularly high risk of CVD would benefit from anticoagulant treatment.

Subgroup analysis revealed that participants who were older than 60 years, female, had a lower BMI level (≤ 30 kg/m^2^), a higher level of eGFR (> 90 ml/min/1.73 m^2^), hypertension and hyperlipidemia and had no co-existed diabetes were prone to have a higher risk of CVD. Participants who had a lower BMI level (≤ 30 kg/m^2^), a higher level of eGFR (> 90 ml/min/1.73 m^2^), and had no co-existed diabetes were prone to have a higher risk of CVD, which appears to be contrary to one would expect as high BMI, low eGFR and diabetes are commonly considered to be in favor of the development of CVD. Considering the universal methodology applied in our study, we speculate that this seemingly paradoxical result may be attributed to the relatively small sample size, whereas such hypothesis needs to be validated in a larger specific population in the future. In addition, due to the non-randomized nature of *post hoc* subgroup analysis applied in this study, these results should be interpreted with caution.

Although a positive association of migraine with CVD has been demonstrated by a previous study (40), the predominant strength of our study lies in that confirmation of such association was based on NHANES database, a nationwide investigation that involves hundreds of thousands of non-institutionalized US civilians and employs standardized study protocols, strict quality control metrics, and specialized technicians who are well-trained to collect and process data, providing a reference for comprehensively estimating the cardiovascular risk among migraine patients across the entire country and highlighting the necessities of targeting migraine as an important risk factor for CVD. In addition, the biggest advantage of using NHANES database is that all samples selected is representative of US citizens residing inside the 50 states and the District of Columbia, which may diminish potential selection bias to the largest extent. However, some limitations still exist in this study. First, the causal relationship between migraine and CVD is hard to establish due to the essence of the cross-sectional design. Therefore, future longitudinal investigations are required to obtain causality inferences about current findings. Second, although we have attempted to search for works in the literature and adjust for potential confounders as much as possible, considering that CVD is a composite of a series of complex and multifaceted diseases, there may still exist some unidentified or unmeasured confounders that may also have a role in the pathogenesis of CVD. In addition, although self-reporting is the most commonly used method to evaluate the presence of CVD (and other covariates) in epidemiological surveys, the self-reported information collected by questionnaires and family interviews may inevitably involve recall bias due to the retrospective nature of self-report measures. Finally, as subtypes and severity of migraine were not explicitly distinguished in the NHANES database, such factors may also impact the difference in cardiovascular risk among migraine individuals.

## Conclusion

In summary, our cross-sectional study revealed a positive association between migraine or severe headache and CVD in a nationally representative US adult population. These findings suggest that migraine should be considered an important risk factor for CVD and that determining whether prevention strategies in migraine patients can reduce the cardiovascular burden is of utmost importance.

## Data availability statement

Publicly available datasets were analyzed in this study. This data can be found here: https://www.cdc.gov/nchs/nhanes/index.htm.

## Ethics statement

The studies involving human participants were reviewed and approved by the NHANES Institutional Review Board [ethical conduct of NHANES 1999–2004 (Protocol#98-12)]. The study procedures were structured in line with the Declaration of Helsinki. The patients/participants provided their written informed consent to participate in this study.

## Author contributions

KW designed the study and completed the experiment together with YM and ML. The first draft was written by KW, and the other authors participated in the revision and polishing of the draft. All authors have approved the final manuscript and declare that all data were generated in-house and that no manuscript mill was used.

## Footnotes

1
https://www.cdc.gov/nchs/nhanes/index.htm

2
http://www.cdc.gov/nchs/data/nhanes/pe.pdf
