# Supplementary material for: Association between migraine and cardiovascular disease: A cross-sectional study
Source: Front Cardiovasc Med. 2022 Nov 24;9:1044465. doi: 10.3389/fcvm.2022.1044465 (PMC9729705; doi:10.3389/fcvm.2022.1044465)
Supplement: Supplementary file 1 [file Table_1.docx]

**Supplementary Table 1 Baseline characteristics of participants excluded and included.**

| Variable | Total | Participants excluded | Participants included | *P* value |
| --- | --- | --- | --- | --- |
| Participants (n) | 15332 | 9640 | 5692 |  |
| Age, years | 50.45 (19.49) | 49.66 (19.61) | 51.77 (19.21) | <0.001 |
| Sex (n, %) |  |  |  | <0.001 |
| Female | 8109 (52.9) | 5216 (54.1) | 2893 (50.8) |  |
| Male | 7223 (47,1) | 4424 (45.9) | 2799 (49.2) |  |
| Education (n, %) |  |  |  | \| 0.193 \| \| --- \| |
| Below high school | 2456 (16.1) | 1505 (15.7) | 951 (16.8) |  |
| High school | 6225 (40.8) | 3923 (40.9) | 2302 (40.6) |  |
| Above high school | 6592 (43.2) | 4174 (43.5) | 2418 (42.6) |  |
| Race (n, %) |  |  |  | \| 0.049 \| \| --- \| |
| Mexican American | 3380 (22.0) | 2186 (22.7) | 1194 (21.0) |  |
| Non-Hispanic Black | 2916 (19.0) | 1833 (19.0) | 1083 (19.0) |  |
| Non-Hispanic White | 7761 (50.6) | 4807 (49.9) | 2954 (51.9) |  |
| Other Hispanic | 699 (4.6) | 436 (4.5) | 263 (4.6) |  |
| Other Race | 576 (3.8) | 378 (3.9) | 198 (3.5) |  |
| BMI, kg/m^2^ | 28.33 (6.24) | 28.31 (6.22) | 28.35 (6.27) | 0.701 |
| Waist, cm | 97.23 (15.08) | 97.17 (14.97) | 97.33 (15.28) | 0.554 |
| TC, mmol/l | 5.27 (1.12) | 5.29 (1.13) | 5.23 (1.10) | 0.002 |
| LDL-C, mmol/l | 3.13 (0.94) | 3.13 (0.93) | 3.14 (0.96) | 0.720 |
| HDL-C, mmol/l | 1.37 (0.41) | 1.38 (0.42) | 1.34 (0.41) | <0.001 |
| Triglyceride, mmol/l | 1.38 (0.96, 2.05) | 1.39 (0.96, 2.07) | 1.37 (0.95, 2.03) | 0.230 |
| Creatinine, umol/l | 70.72 (61.88,88.40) | 70.72 (61.88, 88.40) | 70.72 (61.88, 88.40) | <0.001 |
| eGFR, mL/min/1.73m^2^ | 93.33 (25.19) | 94.72 (25.78) | 90.96 (23.97) | <0.001 |
| Smoke |  |  |  | \| 0.002 \| \| --- \| |
| Never | 7905 (51.7) | 5076 (52.8) | 2829 (49.8) |  |
| Former | 4089 (26.7) | 2516 (26.2) | 1573 (27.7) |  |
| Now | 3303 (21.6) | 2029 (21.1) | 1274 (22.4) |  |
| Alcohol intake (n, %) |  |  |  | 0.434 |
| No | 4242 (32.4) | 2687 (32.7) | 1555 (32.0) |  |
| Yes | 8847 (67.6) | 5540 (67.3) | 3307 (68.0) |  |
| Physical activity |  |  |  | 0.242 |
| No | 7121 (46.5) | 4443 (46.1) | 2678 (47.1) |  |
| Yes | 8190 (53.5) | 5186 (53.9) | 3004 (52.9) |  |
| Poverty |  |  |  | <0.001 |
| No | 12179 (88.0) | 7070 (81.1) | 5109 (100) |  |
| Yes | 1653 (12.0) | 1653 (18.9) | 0 |  |
| Severe headache or migraine (n, %) |  |  |  | 0.087 |
| No | 12275 (80.1) | 7673 (79.7) | 4602 (80.9) |  |
| Yes | 3045 (19.9) | 1955 (20.3) | 1090 (19.1) |  |
| DM (n, %) |  |  |  | 0.969 |
| No | 12487 (86.2) | 7585 (86.2) | 4902 (86.1) |  |
| Yes | 2006 (13.8) | 1217 (13.8) | 789 (13.9) |  |
| Hyperlipidemia |  |  |  | 0.995 |
| No | 4220 (29.7) | 2657 (29.7) | 1563 (29.7) |  |
| Yes | 9989 (70.3) | 6287 (70.3) | 3702 (70.3) |  |
| Hypertension (n, %) |  |  |  | <0.001 |
| No | 8647 (56.4) | 5556 (57.7) | 3091 (54.3) |  |
| Yes | 6679 (43.6) | 4080 (42.3) | 2599 (45.7) |  |
| CVD |  |  |  | 0.005 |
| No | 13439 (87.7) | 8504 (88.2) | 4935 (86.7) |  |
| Yes | 1890 (12.3) | 1133 (11.8) | 757 (13.3) |  |

Data are shown as mean (SD), median (IQR), or n (%). Abbreviations: CVD, cardiovascular disease, BMI, body mass index; DM, diabetes mellitus; TC, total cholesterol; LDL-C, low-density lipoprotein cholesterol; HDL-C, high density leptin cholesterol; eGFR, estimated glomerular ﬁltration rate; SD, standard deviation; IQR, interquartile range.
